# Risk Perception of SARS-CoV-2 Infection and Implementation of Various Protective Measures by Dentists Across Various Countries

**DOI:** 10.3390/ijerph18115848

**Published:** 2021-05-29

**Authors:** Anand Marya, Mohmed Isaqali Karobari, Siddharthan Selvaraj, Abdul Habeeb Adil, Ali A. Assiry, Ali A. Rabaan, Rithvitou Horn, Adith Venugopal, Pietro Messina, Giuseppe Alessandro Scardina

**Affiliations:** 1 Department of Orthodontics, University of Puthisastra, Phnom Penh 12211, Cambodia; amarya@puthisastra.edu.kh; 2 Department of Orthodontics, Saveetha Dental College, Saveetha Institute of Medical and Technical Sciences, Chennai 600077, India; avenugopal@puthisastra.edu.kh; 3 Conservative Dentistry Unit, School of Dental Sciences, Universiti Sains Malaysia Health Campus, Kubang Kerian 16150, Malaysia; 4 Faculty of Medicine, UniSZA, Kuala Terengganu, Terengganu 20400, Malaysia; sidzcristiano@gmail.com; 5 Department of Community Dentistry, School of Dental Sciences, Universiti Sains Malaysia Health Campus, Kubang Kerian 16150, Malaysia; adilhabeeb@gmail.com; 6 Preventive Dental Science Department, Faculty of Dentistry, Najran University, Najran 55461, Saudi Arabia; assirypedo@gmail.com; 7 Molecular Diagnostic Laboratory, Johns Hopkins Aramco Healthcare, Dhahran 34465, Saudi Arabia; arabaan@gmail.com; 8 Faculty of dentistry, University of Puthisastra, Phnom Penh 12211, Cambodia; hrithvitou@puthisastra.edu.kh; 9 Department of Surgical, Oncological and Stomatological Disciplines, University of Palermo, 90133 Palermo, Italy; pietro.messina01@unipa.it

**Keywords:** COVID-19, infection, dentists, risk, protective

## Abstract

Objective: Healthcare workers in general are at a high risk of potential infections with COVID-19, especially those who work with aerosol generating procedures. Dentists fall in this category, as not only do they operate with aerosol generating procedures but also operate within a face-to-face contact area. Methods: A structured self-administered questionnaire was developed at Najran University and provided to the participants for data collection. The data collected included information on risk perception and incorporation of measures for protection against COVID-19 to gauge the attitude of dentists during this period. Also, clinical implementation of various protective measures was reviewed. Results: Of the *n* = 322 dentists that answered the questions, 50% were general dentists and 28.9% were dentists working at specialist clinics, while the remaining 21.1% of dentists were employed in academic institutions. Among the newer additions to the clinic, 36.3% of dentists answered that they had added atomizers to their practices, followed by 26.4% of dentists that had incorporated the use of UV lamps for sterilization. We found that 18.9% dentists were using HEPA filters in their clinics, while 9.9% of dentists were making use of fumigation devices to control the risk of infection. One-way ANOVA was also carried out to demonstrate that there was a statistically significant difference (*p* = 0.049) between groups of dentists utilizing HEPA filters, UV lamps, atomizers, and fumigation devices to prevent the spread of SARS-CoV2 across their workplaces. Conclusion: Dentists are aware of recently updated knowledge about the modes of transmission of COVID-19 and the recommended infection control measures in dental settings. A better understanding of the situation and methods to prevent it will ensure that the dental community is able to provide healthcare services to patients during the pandemic.

## 1. Introduction

The COVID-19 pandemic originated in Wuhan in December 2019 and was declared a public health emergency by the World Health Organization in the year 2020 [1]. Initially it was identified in the bronchoalveolar fluid samples from patients suffering from pneumonial infection and it was designated the name: the novel coronavirus. Subsequently the International Committee on Taxonomy of viruses announced that the virus would be known as SARS-CoV2 (severe acute respiratory syndrome coronavirus 2) as it genetically resembled the coronavirus subtype from the SARS epidemic that occurred in the year 2003 [2]. Thereafter the WHO decided to rename the virus to COVID-19, and it has been known by this name thereafter. The past year has seen the rise of infections across the globe and there is no region left which has not been impacted by this pandemic. Healthcare workers in general are at a high risk of potential infections with COVID-19, especially those who work with aerosol-generating procedures. Dentists fall in this category, as not only do they operate with aerosol-generating procedures but also operate within a face-to-face contact area (0.5 m) [3].

### 1.1. Modes of Transmission

Several studies conducted on SARS-CoV2 transmission have demonstrated that the spread occurs mostly through respiratory droplets and direct contact with an infected individual. When an infected individual is in proximity with a non-infected individual, they can transmit the infection via oral or nasal droplets [4]. There are also other studies that have reported the transmission of infection via the ocular surface, pass through the nasolacrimal ducts, and then enter the respiratory system of non-infected individuals [5]. For these purposes, infection control guidelines have been set down that include washing hands with soap for a minimum of 20 s, use of alcohol-based rubs or isopropanol [6].

### 1.2. Transmission Risk in Dental Offices

In dental clinics, COVID-19 transmission is expected via aerosol generating procedures such as the use of high-speed handpieces or ultrasonic scalers [7]. Contaminated surfaces in a dental clinic may also act as potential infection-transmitting agents. During dental treatment procedures aerosols are generated by the patients, the waterlines, and even the instruments in use [8,9]. Patients may transmit infections to dentists or assistants who operate in proximity by means of aerosols carrying micro-organisms. Waterlines (DUWLs) may transfer infectious micro-organisms to the patient’s oral cavity during treatment procedures. Instruments such as high-speed handpieces, ultrasonic scalers, and air-water syringes cause aerosol generation that may help spread infections. To minimize the risk of infections from dental aerosols there are infection control guidelines that lay emphasis on the use of personal protective equipment, i.e., PPE which include masks, eye wear, and gloves [10]. These guidelines are particularly useful during the pandemic as the incubation period of COVID-19 is up to 14 days which means that even asymptomatic cases can transmit infection to others [11].

### 1.3. The Current Scenario during the Second Wave

During the first period of the COVID-19 pandemic the risk for dental healthcare professionals was particularly high and there were many infected cases initially. The high mortality rate among dentists and healthcare workers prompted the healthcare bodies to provide recommendations for safe treatment. These measures included the use of telephonic triaging to screen patients before their appointments, temperature screening at the clinic entry, spaced seating, hand disinfection, and use of disposable instruments [12]. Unfortunately, the risk of infection transmission is still high during the second wave currently happening around the world, particularly because of many asymptomatic patients. This risk will continue to be high until a large part of the global population is vaccinated. This is the reason why dentists must continue to employ various protective measures to ensure there is prevention against COVID-19 infections.

This study was conducted with an aim of analyzing the risk of infection as perceived by dentists and the efforts made by them to prevent the spread of this highly transmissible viral infection.

## 2. Methods

### 2.1. Site and Settings

The study was carried out with approval from institutional ethical committee at Faculty of Dentistry, Najran University with the assigned ethical approval number: (2021/0022). A structured self-administered questionnaire was developed at Najran University and provided to the participants for data collection. This study aimed to analyze the various protective measures that have been employed by dentists in their clinics for prevention against SARS-CoV2 infections. It also aims to provide an overview of the current situation in terms of dentist’s attitudes towards the pandemic.

### 2.2. Questionnaire

An online questionnaire was developed using Google survey to collect demographic data and study the risk perception of dentists. The data collected included information on risk perception and incorporation of measures for protection against COVID-19 to gauge the attitude of dentists during this period. The questionnaire was validated by distributing it among 20 dentists (content experts) in the institute initially and following validation it was distributed online among dentists from each of the countries via emails containing links to the Google survey. No identifying data were collected at any point in the study. Also, a discretion statement was included in the questionnaire for the participants.

### 2.3. Subjects

**Figure 1:**
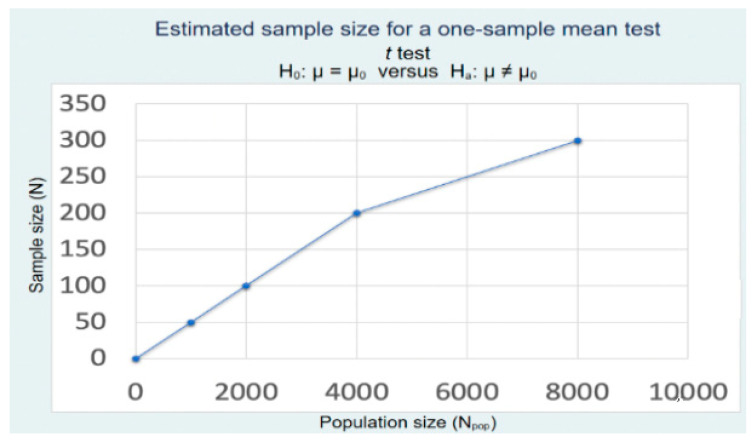
Sample size estimation for dentists. Parameters: α = 0.05, 1 – β = 0.8, δ = 0.29, µ_0_ = 0.01, µ_a_ = 0.3, σ = 1.

The dental practitioners were based in India, Malaysia, Saudi Arabia, Thailand, Cambodia, and Italy. Licensed dental practitioners with work experience of more than a year were selected for this study. The researchers provided the online links to the questionnaire making use of Google survey and non-qualified dental professionals, and dentistry students were excluded from the study. Sample size analysis was carried out in this study to ascertain that a study sample of 300 dentists would be optimal to achieve the research objectives of the study. As can be seen from the results of the sample size analysis, a study sample of approximately 300 dentists is enough to represent a dental population of the size of 8000 dentists, thus making the outcome of the study successful (Figure 1). The one-sided hypothesis test also asserted that the target sample for the sample taken for the study was enough to get an optimal outcome.

### 2.4. Bias

The questionnaires were designed based on previous evidence-based research that enabled participants in the study to answer questions related to protective practices followed in their clinics. Other factors that were considered including planning the content, questionnaire layout and order, piloting, response rate, and considering the content validity of the questions.

### 2.5. Statistics

A descriptive analysis, sample size calculation, and significance were identified using STATA/IC 16.1 statistical software (StataCorp, College Station, TX, USA).

## 3. Results

The online questionnaire contained demographic information such as qualifications, type pf job- general practice, specialist practice, or an academic job. Data was collected from participating dentists regarding their clinical protective practices during the pandemic and an effort was made to compile all of the results and correlate them with findings from other evidence-based studies. Out of a total of *n* = 350 dental practitioners that were sent the online questionnaire, *n* = 322 dentists responded by completing and answering all questions that were asked. Of the *n* = 322 dentists that answered the questions, 50% were general dentists and 28.9% were dentists working at specialist clinics while the remaining 21.1% dentists were employed in academic institutions. While 46.9% respondents replied that their areas had no lockdown, there were another 48.4% dentists that responded by recording that their practices were still in areas under partial lockdowns. Of our sample, 39.1% dentists were still apprehensive about working under stressful conditions and perceived the situation as high risk, while 46% dentists felt that they had got accustomed to the situation and it was acceptable to work as they saw the current scenario as a low-risk situation. Out of the total number of respondents, a high percentage of 88.5% dentists were using PPE (personnel protective equipment) to deliver treatment to their patients while the remaining dentists replied non-affirmatively, as they were either not working or in lockdown.

During the pandemic, tele dentistry and online consultations gained a lot of prominence and this was reflected in the results, as 80.1% of dentists were found have been utilizing online consultations to follow-up or consult their patients, while 7.1% of dentists replied in the negative. When asked about the patient’s threat perception, 32% of dentists felt that the patients would feel less worried after 3 to 6 months, while 15.2% of dentists felt that it may take up to a year for the patients to feel less threatened by the risk of COVID-19. Of our sample, 46.9% of dentists felt that more than 25% to 50% of their patients were concerned about their appointments being cancelled, while 38.8% of dentists responded that only very few of their patients complained about cancelled appointments. Regarding allowing family members/companions, 76.7% of dentists replied that they were allowing companions with the patients, while 23.3% of dentists answered in the negative.

Since tele dentistry has proven to be immensely valuable during the pandemic period, 87% dentists said that they would continue to use online consultations even after the situation goes back to normal. A high percentage of respondents, i.e., 92.9% dentists answered that they had been consulting new patients during the pandemic period, while 7.1% did not consult any new patients.

**Figure 2:**
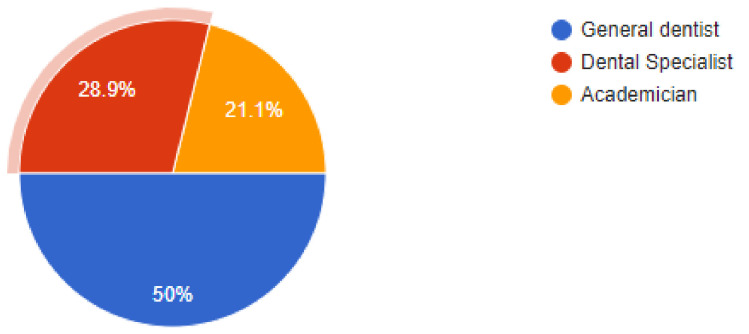
Percentage division by Profession of dentists.

**Figure 3:**
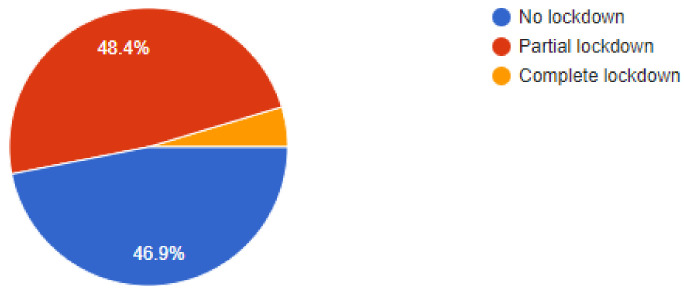
Current state of Lockdown.

**Figure 4:**
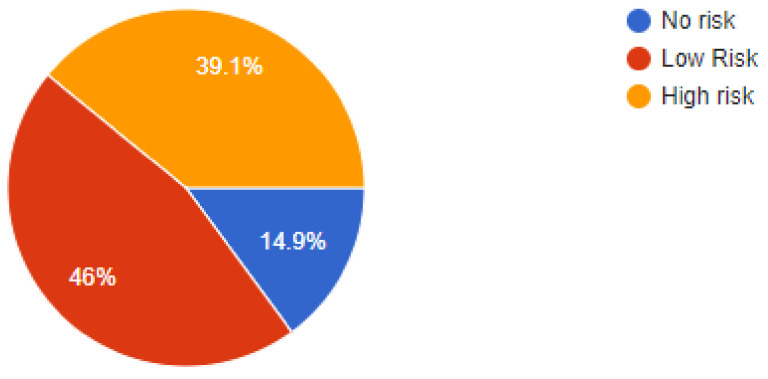
Risk perception of dentists.

**Figure 5:**
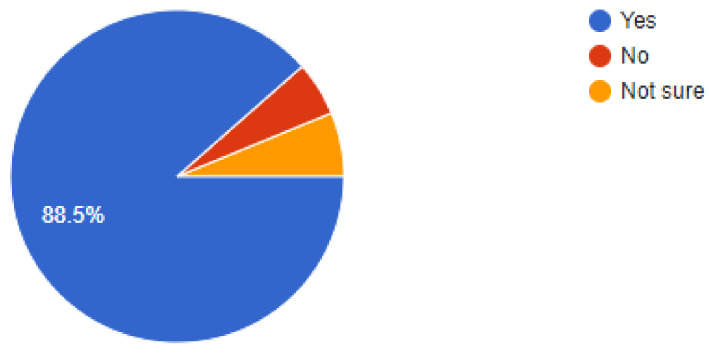
Dentists in agreement to wear PPE and practice.

**Figure 6:**
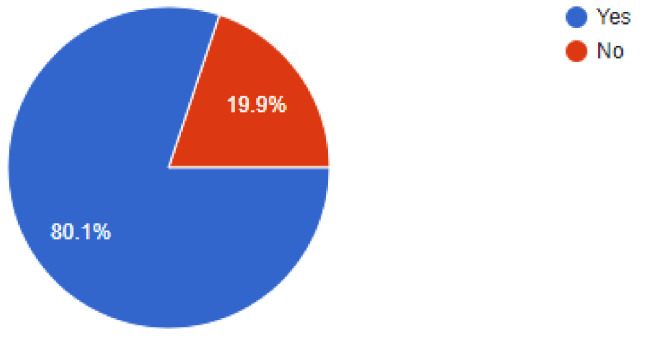
Carrying online consultations or not?

**Figure 7:**
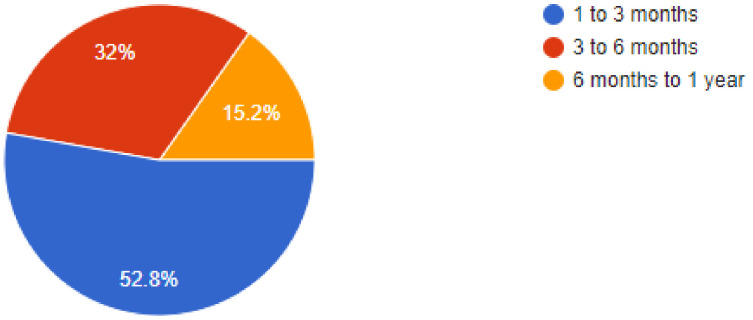
How long do you think patients will be affected by the virus threat?

**Figure 8:**
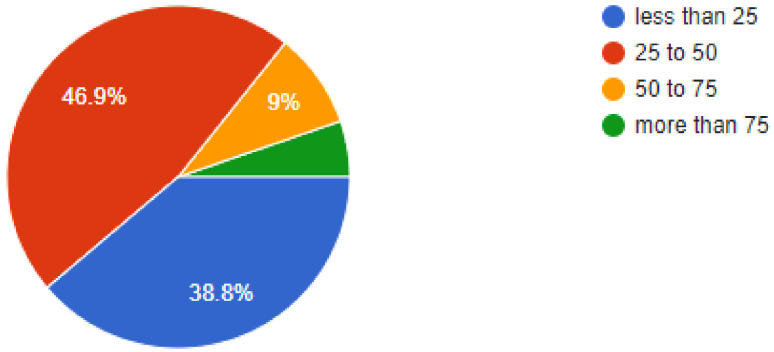
What percentage of patients have problems with existing schedules being cancelled?

**Figure 9:**
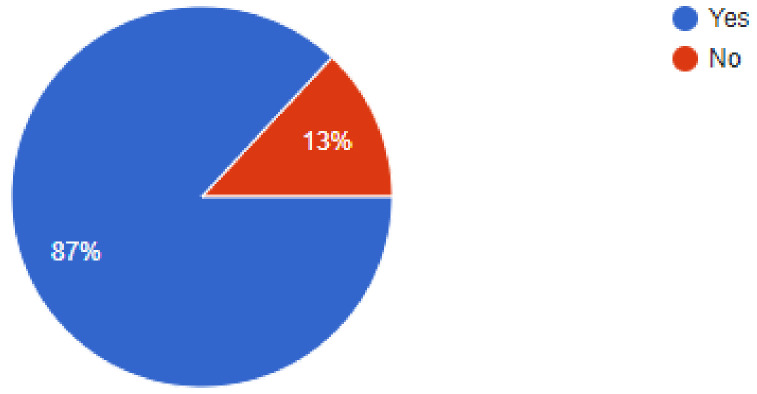
Are you still using virtual consultations, and will you continue to do so?

**Figure 10:**
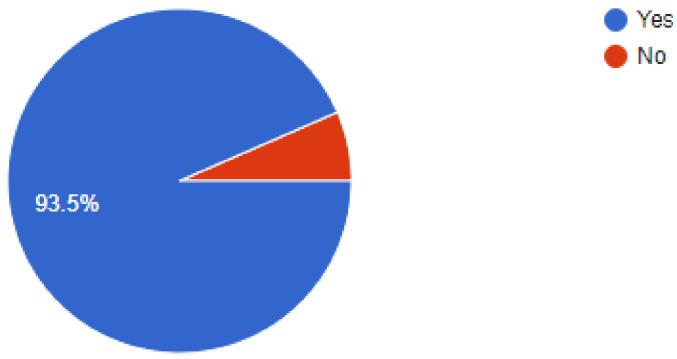
Have you added any measures for disinfection?

**Figure 11:**
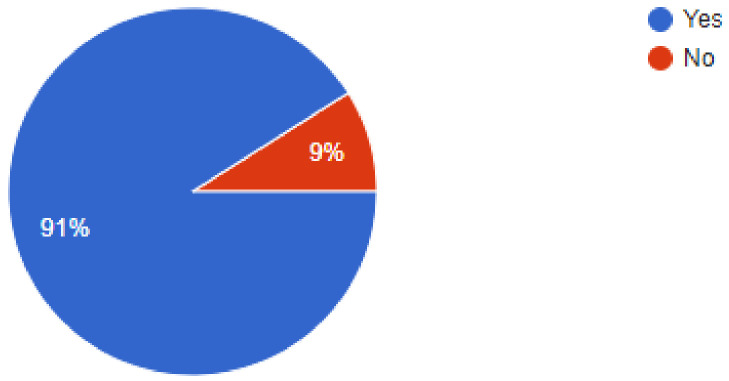
Do you strictly implement the 1-meter distance for seating patients in your clinic?

**Figure 12:**
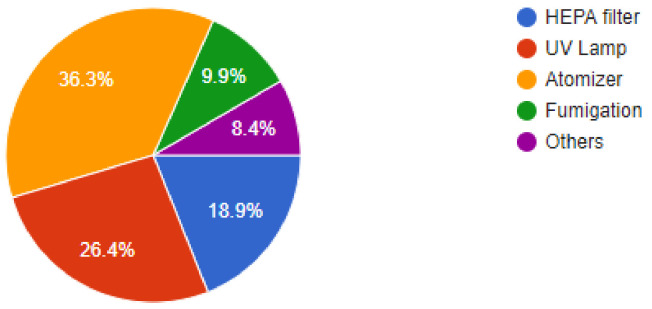
Are you using any of the following?

**Figure 13:**
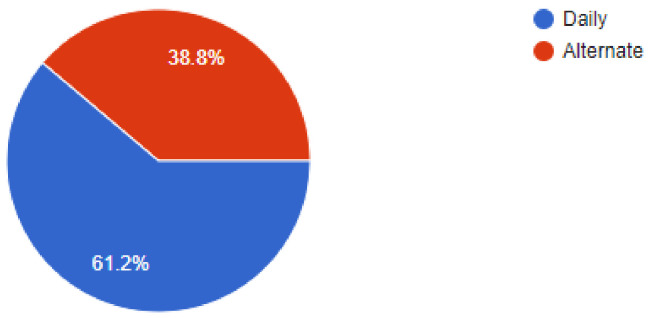
On what basis is your clinic making use of staff?

**Figure 14:**
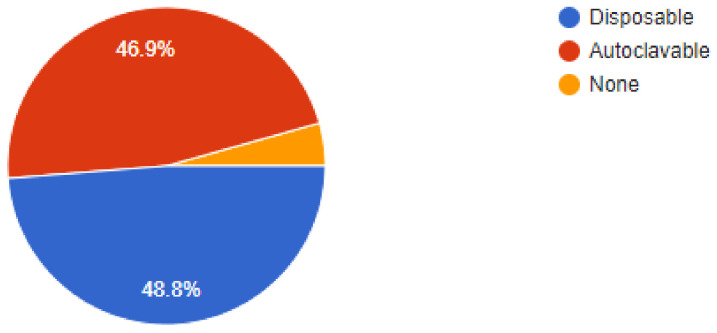
Have you added more instruments? If so, are they disposable or autoclavable?

**Figure 15:**
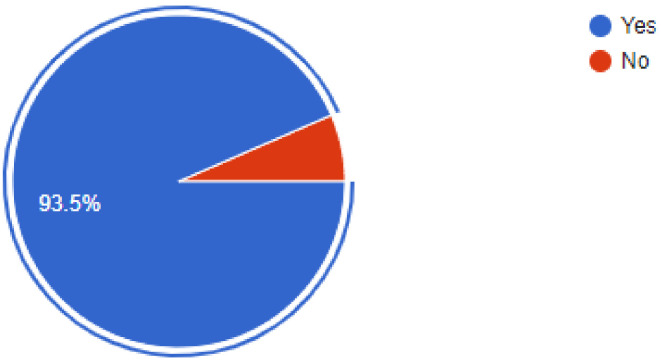
Will you continue to employ these measures once you and your staff are vaccinated?

The importance of newer protective measures could be seen in the responses of the dentists, as 93.5% of dentists were found to have added devices or measures to their clinics to help lower the risk of SARS-CoV2 infections. Of our sample, 91% of dentists were found to have been following a 1-meter spaced seating arrangement in the clinics, which has been recommended by healthcare regulatory bodies around the world. Among the newer additions to the clinic, 36.3% of dentists answered that they had added atomizers to their practices, followed by 26.4% of dentists that had incorporated the use of UV lamps for sterilization. Of our sample, 18.9% of dentists were using HEPA filters in their clinics, while 9.9% of dentists were making use of fumigation devices to control the risk of infection. Most of the dentists, i.e., 61.2% were still employing their staff daily, while 38.8% of dentists had asked their staff to come to work only on alternate days for various reasons. Of our sample, 48.8% of dentists had added disposable instruments to their clinical inventory, while 46.9% of dentists had added more autoclavable instruments. In our sample, 93.5% of dentists answered that they would continue to employ the measures newly added to their clinics for the foreseeable future, even after the pandemic is under control (Figure 2, Figure 3, Figure 4, Figure 5, Figure 6, Figure 7, Figure 8, Figure 9, Figure 10, Figure 11, Figure 12, Figure 13, Figure 14 and Figure 15).

One-way ANOVA was carried out to evaluate any statistically significant differences between the three groups of dentists who responded in this study, and it was seen that the result was significant, i.e., *p* = 0.049. One-way ANOVA was done to understand the difference between measures implemented by general dentists, specialist dentists, and academicians with the significance set at 0.05. It was seen from the results obtained that there was a statistically significant difference between the autoclavable, and disposable measures adopted by the different groups of dentists (Table 1). One-way ANOVA was also carried out to find out whether there were differences in the measures adopted by these groups. Again, there was a statistically significant difference between groups of dentists utilizing HEPA filters, UV lamps, atomizers, and fumigation devices to prevent the spread of SARS-CoV2 across their workplaces (Table 2).

One-way ANOVA was also used to determine if there was any significant difference in the implementation of preventive measures among dentists from different countries. It was seen that there were significant differences between participating dentists from different countries in terms of implementation of the 1-meter spaced seating in their dental offices for various reasons (*p* < 0.05) (Table 3). There was also a significant difference when it came to various devices being used to prevent the transmission of SARS-CoV-2 in dental offices (*p* < 0.05) (Table 4).

## 4. Discussion

This study used data collected from practicing dentists and academicians as the baseline to assess the infection threat perception and their attitude towards the pandemic. A conscious decision was made to include dental academicians in the study as well, because previously conducted studies had shown that they had poorer knowledge of the spread of the infection [13]. One of the major strengths of this study was the sample size and diversity of the respondent’s countries which allowed us to understand and analyze the differences in the attitude of dentists across a multicultural environment. The rapidly progressive nature of this pandemic has led to our findings being time relevant, and therefore our aim was to focus on a broader group and provide useful insights. In the present study, dentists demonstrated a higher knowledge of COVID-19 transmission and associated measures compared to studies conducted solely across single countries [14]. This may be attributed to the fact that there is a difference in information being made available to dentists across different regions and may have an impact on how safety is being practiced. It was seen that a high percentage of dentists were apprehensive of working under the threat of an infection because the vaccination process is still underway in many countries and may take some time to accomplish [15].

Incorporation of tele dentistry into practices has helped contain the spread of the virus across the dental community, and this was reflected in the results of the study which matched those of previously conducted research [16,17]. Telephonic triaging has also been one of the more recently implemented measures during the pandemic used by dental clinics to ascertain whether some patients maybe showing symptoms and to move their appointments to another time [18]. Although there have been a lot of patients who have complained about cancelled appointments during this period, the lack of spread across dental clinics and hospitals in these regions should reimpose faith among patients that these steps were for their good. Most dentists interviewed responded that they had added newer measures at their clinics to help check the spread of this virus, and these included atomizers, UV lamps, HEPA filters, etc., which have been recommended by various health regulatory bodies globally [19,20]. It has been shown in previous studies that air purifiers with HEPA H12 class filters are much more protective and effective compared to simple purifiers at aerosol removal during dental procedures [21]. Purifiers with simple F6 filters were shown to be only 53% effective at aerosol removal so even the choice of purifier and filters must be very precise. UV lamps and atomizers were found to be very popular during the study and it has been demonstrated previously that irradiation with ultraviolet light is effective against coronaviruses [22,23]. UV lamps come at a much more economical cost compared to the other options and have been effective at sterilizing both contaminated surfaces and bioaerosols.

The findings of the study demonstrated that a vast majority of dentists had added new autoclavable and disposable instruments to their clinics to minimize the risk of infection via the use of instruments [24] (Table 1). A major positive outcome of this study was that respondents answered that they would continue to implement the newer infection control measures even after the pandemic has subsided [25]. This should ensure a much safer environment for patients to be treated in for the long-term foreseeable future and keep infection risks to a minimum.

## 5. Conclusions

To ensure the safety of the dental staff and personnel during the pandemic, various health regulatory bodies have set down guidelines and recommendations. It is essential to educate all staff in the clinic regarding infection control measures to ensure that there is a check on disease progression. Patients who visit the clinics must also be educated prior to their visit on infection control measures being implemented, preventive methods, and any newer hygiene guidelines. A better understanding of the situation and methods to prevent it will ensure that the dental community is able to provide healthcare services to patients during the pandemic.

## 6. Limitations

One of the limitations of this paper is the cross-sectional design which cannot evaluate the changes in perception, as well as the attitude of dentists during the entire pandemic period, as these may change over time. While convenience sampling maybe seen as a statistical limitation in many cases, in this study it was the only feasible method to gather data from different regions. The number of responses were gathered by sending reminders, which may be low for a study of this magnitude, but previous literature has shown that health professionals usually respond in low numbers to online surveys [26]. However, the data collected from ethnically and culturally diverse environments should ensure the generalizability of the results obtained from this study.

## 7. Recommendations

Dental healthcare has been significantly affected by the COVID-19 pandemic. Limiting dental care to emergency treatment and postponing elective procedures not only challenged the current treatment protocols but also resulted in a significant financial loss to many dental practices. The risk of COVID-19 infection to the dental care provider and potential to infect patients during emergency dental treatment are significant.

Based on the findings of this study we would like to offer the following recommendations:

1. More efforts need to be directed at evidence-based clinical research to establish COVID-19 guidelines for each geographic location.
2. The organization of educational programs for dentists to create more awareness about the infection risks in the clinics and preventive measures to adopt.
3. Continuous evaluation of preventive measures to ensure that these are not adopted on a short-term basis and neglected thereafter.

## Figures and Tables

**Table 1:** Differences between autoclavable and disposable measures added by different groups of dentists.

Have You Added Any Measures for Disinfection?	None	Disposable	Autoclave	Total	One-Way ANOVA
	*n* (%)	*n* (%)	*n* (%)	*n* (%)	
Academician	1 (1.8)	32 (56.1)	24 (42.1)	57 (100.0)	0.049
General dentist	5 (4.6)	53 (49.1)	50 (46.3)	108 (100.0)	
Dental specialist	2 (2.9)	33 (48.5)	33 (48.5)	68 (100.0)	

*p* < 0.05.

**Table 2:** Differences between various preventive modalities added to the clinics with different groups of dentists.

Profession	HEPA Filter	UV Lamp	Atomizer	Fumigation	Total
	*n* (%)	*n* (%)	*n* (%)	*n* (%)	*n* (%)
General dentist	36 (25.4) ^b^	42 (29.6) ^a^	50 (35.2) ^b^	14 (9.9) ^b^	142 (100.0)
Dental specialist	19 (21.8) ^b^	24 (27.6) ^a^	37 (37.9) ^b^	11 (12.6) ^b^	87 (100.0)
Academician	6 (9.1) ^a^	19 (28.8) ^a^	34 (51.5) ^a^	7 (10.6) ^a^	66 (100)

Significant level One-way ANOVA = 0.05. ^a^ and ^b^ represent values that are significantly different statistically.

**Table 3:** Differences between dentists from various countries for the implementation of the 1-meter spaced seating in their clinics.

			13. Have You Incorporated the 1-Meter Distance for Seating in Your Clinic?		Total	*p*-Value
			No	Yes		
1. Location of Practice	Cambodia	*n*	2	7	9	0.000
		%	22.2%	77.8%	100.0%	
	India	*n*	2	101	103	
		%	1.9%	98.1%	100.0%	
	Malaysia	*n*	2	99	101	
		%	2.0%	98.0%	100.0%	
	Qatar	*n*	12	25	37	
		%	32.4%	67.6%	100.0%	
	Saudi	*n*	11	60	71	
		%	15.5%	84.5%	100.0%	
	Thailand	*n*	0	1	1	
		%	0.0%	100.0%	100.0%	
	Italy	*n*	0	50	50	
		%	0.0%	100%	100%	
Total		*n*	29	293	322	
		%	9.0%	91.0%	100.0%	

*p* < 0.05.

**Table 4:** Differences between the implementation of protective measures/devices in dental clinics in different countries.

Crosstab									*p*-Value
			14.Are You Using Any of the Following?					Total	
			Atomizer	Fumigation	HEPA Filter	Others	UV Lamp		
1. Location of Practice	Cambodia	*n*	1	0	0	3	5	9	0.003
		%	11.1%	0.0%	0.0%	33.3%	55.6%	100.0%	
	India	*n*	22	16	13	4	48	103	
		%	21.4%	15.5%	12.6%	3.9%	46.6%	100.0%	
	Malaysia	*n*	74	8	6	6	7	101	
		%	73.3%	7.9%	5.9%	5.9%	6.9%	100.0%	
	Qatar	*n*	5	0	22	2	8	37	
		%	13.5%	0.0%	59.5%	5.4%	21.6%	100.0%	
	Saudi	*n*	15	8	19	12	17	71	
		%	21.1%	11.3%	26.8%	16.9%	23.9%	100.0%	
	Thailand	*n*	0	0	1	0	0	1	
		%	0.0%	0.0%	100.0%	0.0%	0.0%	100.0%	
	Italy	*n*	5	0	20	15	10	50	
		%	10%	0.0%	40%	30%	20%	100%	
Total		*n*	122	32	61	27	85	322	
		%	36.4%	9.9%	18.9%	8.4%	26.4%	100.0%	

*p* < 0.05.

## Data Availability

The data used to support the findings of this study are available from the corresponding authors upon request.
